# Uncovering Key Parameters in Perfluorosulfonic Acid (PFSA) Membrane Fuel Cells to Enhance Performance

**DOI:** 10.3390/membranes15030065

**Published:** 2025-02-20

**Authors:** Valdecir A. Paganin, Alan M. P. Sakita, Thiago Lopes, Edson A. Ticianelli, Joelma Perez

**Affiliations:** 1 São Carlos Institute of Chemistry, University of São Paulo, São Paulo 13560-970, Brazil; vpaganin@iqsc.usp.br (V.A.P.); ampsakita@usp.br (A.M.P.S.); edsont@iqsc.usp.br (E.A.T.); 2 Research Centre for Greenhouse Gas Innovation—RCGI and Escola Politecnica, University of São Paulo, Av. Professor Mello Moraes, São Paulo 13560-970, Brazil; thiago_lopes@usp.br

**Keywords:** PFSA membranes, hot-pressing, cell tightening, PEMFCs, cell performance, pressure homogenizer, MEA preparation, hydrogen desorption

## Abstract

The conversion of chemical energy to electricity in proton exchange membrane fuel cells (PEMFCs) is essential for replacing fossil fuel engines and achieving net-zero CO_2_ emissions. In the pursuit of more efficient PEMFCs, certain often-overlooked parameters significantly influence cell performance by either weakening the interaction between the catalytic layer (CL) and the membrane or restricting gas access to the CL. This study examines the effects of cell tightening and hot-pressing conditions on three similar-thickness perfluorosulfonic acid (PFSA) membranes: Aquivion^®^, Fumapem, and Nafion^®^. The results reveal that the hot-pressing method employing higher pressure and a lower temperature (125C method) yields lower fuel cell performance compared to the method utilizing a higher temperature and lower pressure (145C method). Furthermore, incorporating cellulose paper as a pressure homogenizer in the MEA preparation setup significantly improved current density by approximately 2.5 times compared to the traditional assembly method. Cyclic voltammetry with Ar-feed in the cathode showed that all prepared MEAs exhibited a similar platinum surface area; however, MEAs pressed at higher temperatures displayed slightly lower hydrogen desorption charge values. The torque applied to the bolts does not show a consistent trend in fuel cell performance, but optimal torque values can enhance PEMFC performance under certain conditions.

## 1. Introduction

Pursuing net-zero CO_2_ emissions has driven the study and optimization of environmentally friendly energy storage and conversion technologies. Among the most prominent options, proton exchange membrane fuel cells (PEMFCs) have garnered significant attention for both transportation and stationary applications, primarily due to their use of green fuels and their minimal (or even zero) release of greenhouse gases during operation [1]. The environmental benefits of PEMFCs depend significantly on the hydrogen source. When derived from fossil fuels (gray hydrogen), substantial carbon emissions persist, undermining sustainability goals. Therefore, priority should be given to green hydrogen, produced via water electrolysis powered by renewable energy [2].

The energy conversion core of the PEMFCs is the membrane electrode assembly (MEA), which consists of a pair of gas diffusion electrodes (GDEs, anode and cathode) containing an appropriate catalyst, in close contact with a proton exchange membrane (PEM) that serves as the cell electrolyte. While the catalysts loaded onto the GDEs play a critical role in enhancing cell performance and lifespan, other less conventional parameters influence their behavior.

For instance, platinum nanoparticles supported on carbon (Pt/C) are well-studied catalysts for the hydrogen oxidation reaction (HOR) and the oxygen reduction reaction (ORR), representing the state-of-the-art catalysts for both processes [3]. However, despite their catalytic activity in half-cell studies often showing consistent behavior as reported in the literature, the performance of Pt/C catalysts can vary significantly when employed in a single cells. This variation is closely linked to factors such as membrane characteristics, fuel-cell mounting parameters, MEA fabrication methods, and GDE preparation.

Regarding the membrane, it is well-documented that thinner membranes are the preferred choice for achieving a higher power output due to their lower resistance, providing a significant advantage compared to thicker membranes [4]. Additionally, from an economic standpoint, thinner membranes require less perfluorosulfonic polymer, one of the most expensive components of fuel cells [5]. On the other hand, under the varying operational conditions of a PEMFC, such as hot/cold or wet/dry cycles, thinner membranes are more prone to failure, leading to increased gas permeability. In contrast, thicker membranes exhibit greater structural stability, reducing the likelihood of such issues [6].

However, thickness is not the only parameter that impacts membrane characteristics. The size of the perfluorosulfonic acid (PFSA) side chain and the equivalent weight (EW) of the polymer also influence critical properties such as water uptake, thermal stability, and gas permeability [7], all of which significantly affect fuel cell performance [8]. Typical PFSA ionomers consist of a PTFE backbone with perfluoropolyether side chains functionalized with sulfonic acid (–SO_3_H) groups at their ends. While the main chain is mainly hydrophobic, the side chain domains form hydrophilic domains that swell upon water absorption due to interactions with the –SO_3_H groups, facilitating the proton conduction.

For instance, Shinoda et al. [9] demonstrated that the EW of PFSA membranes impacts hydrogen permeation, with polymers of a lower EW and low crystallinity exhibiting reduced H_2_ permeability due to the tortuous network structure of their aqueous domains. This reduced permeability of H_2_ (as well as O_2_ or air) is beneficial, as excessive gas permeability can detrimentally affect fuel cell performance by lowering the open circuit potential (OCP) and accelerating degradation [10]. Similarly, shorter PFSA side chains also exhibit lower gas permeability, as demonstrated by Patel et al. [7].

On the other hand, various methods can be employed for preparing the MEA: directly coating the catalyst ink (a mixture of catalyst, ionomer, and solvent) onto the membrane; hot-pressing the gas diffusion electrode (GDE) to integrate it with the membrane; or simply placing the electrodes against the membrane during the cell assembly process, a technique also known as non-hot-pressing. Each method influences fuel cell performance by improving the contact between the GDE and the membrane or enhancing catalyst utilization [11]. Among these, hot-pressing is one of the most widely used methods due to its simplicity, allowing the catalytic layer to be deposited directly onto the GDE substrate and subsequently integrated with the membrane by pressing the electrode at elevated temperatures (<160 °C). These methods for preparing the MEA were systematically investigated by Li et al. [12], who demonstrated that the hot-pressing of coated substrates onto the membrane exhibits comparable performance to directly coating the membrane. However, the open circuit voltage and overall cell performance can be severely impacted depending on the hot-pressing parameters and membrane characteristics.

Regarding hot-pressing parameters, two key variables influence the hot-pressing process of MEAs: temperature and pressure. A certain temperature is required to soften the polymer chains, while pressure ensures the uniform distribution of heat and the proper adhesion of the GDE to the membrane. Optimizing these parameters is crucial for enhancing PEMFC performance.

Temperature plays a critical role, as PFSA membranes are known to be susceptible to degradation at temperatures that are significantly higher than their first glass transition temperature (T_g_). Kawano et al. [13] reported the loss of sulfonic acid groups in the temperature range of 290–400 °C, as determined by thermogravimetric analysis. This finding is consistent with earlier observations by Savinel et al. [14], who reported similar degradation behavior. Despite this, two distinct perspectives are commonly observed regarding hot-pressing. In the literature, one perspective suggests that the hot-pressing temperature should be around or slightly above the 1st T_g_ of PFSA polymers [15,16]. However, several studies have reported improved PEMFC performance when MEAs are pressed at temperatures significantly below 1st Tg [17,18].

In contrast, the applied pressure is not determined based on any specific figure of merit related to the membrane characteristics, such as Young’s modulus. For example, Bender et al. [19], using different Nafion membranes with a compression force range of 14–25 kg cm^−2^ and temperatures of either 120 or 125 °C, around the membrane T_g_, revealed that higher pressing temperatures (125 °C) damage the membrane, creating irregularities that increase hydrogen permeability over time, thereby negatively affecting performance. Nonetheless, while lower pressures resulted in higher open-circuit voltages, they also led to lower cell performance in terms of current density at a given potential and maximum power density compared to MEAs prepared with 25 kg cm^−2^.

Another critical factor arises during the mounting of the MEA in the fuel cell. The compression of the GDE, controlled by the separator-to-GDE thickness ratio or by the torque applied when tightening the fuel cell bolts, is an additional variable that impacts gas transport through the GDE to the catalytic layer, cell resistance and pore distribution within the GDE components [20,21]. Harris et al. [21] evaluated the cell compression of non-hot-pressed MEAs by adjusting the separator thickness, demonstrating that optimal performance is achieved at approximately 14% GDE compression. These authors also found that excessive GDE compression exacerbates cathode flooding, as indicated by the presence of diffusional currents in the polarization curves and an increase in the low-frequency arc observed in electrochemical impedance spectroscopy (EIS). Similarly, the torque applied to tighten the fuel cell bolts produced effects that were consistent with those observed by Harris et al., with over-compression having deleterious impacts on fuel cell performance [22,23].

As highlighted, these often-overlooked parameters can play a pivotal role in improving PEMFC performance. However, many authors frequently neglect them, likely due to a primary focus on addressing the challenges of developing more stable and cost-effective catalysts. In this work, we investigate the effects of membrane characteristics, hot-pressing conditions, and compression rates on the performance of PEMFCs constructed with commercially available Pt-based electrodes. By systematically studying these parameters, this work provides a comprehensive understanding of how the interplay between these factors influences fuel cell characteristics and an accurate choice could improve their performance.

## 2. Materials and Methods

### 2.1. Materials

The commercial gas diffusion electrodes containing Pt and the PFSA ionomer were purchased from Alfa Aesar (H₂ electrode/reformate electrode, 045,372 Alfa Aesar, UK). Nafion^®^ 211 and 212 membranes were obtained from Ion Power Tyrone, PA, USA. Fumapem F-930 and F-950 membranes were supplied from Fuel Cell Store Bryan, TX, USA, while the Aquivion^®^ E98-05S membrane was purchased from Sigma-Aldrich. Table 1 summarizes the difference between each studied membrane.

### 2.2. Membrane Pre-Treatment

Nafion^®^ membranes in their dry form were activated by immersion in 3 wt.% H_2_O_2_ at 80 °C for one hour [24]. After this treatment, the membranes were rinsed to remove any traces of H_2_O₂, followed by a similar procedure in 0.5 mol L⁻^1^ of H_2_SO_4_ at the same temperature. Fumapem membranes were activated by immersing them in 5 wt.% H_2_SO_4_ at 80 °C for 12 h. Aquivion^®^ membranes were activated by immersion in 1 mol L⁻^1^ of H_2_SO_4_ at 80 °C for one hour. After activation, all membranes were thoroughly rinsed with Milli-Q water until the rinse water reached a neutral pH. Each pre-treatment followed the manufacturer’s recommended procedure to ensure optimal membrane performance.

### 2.3. Membrane Electrode Assembly Preparation

**Figure 1:**
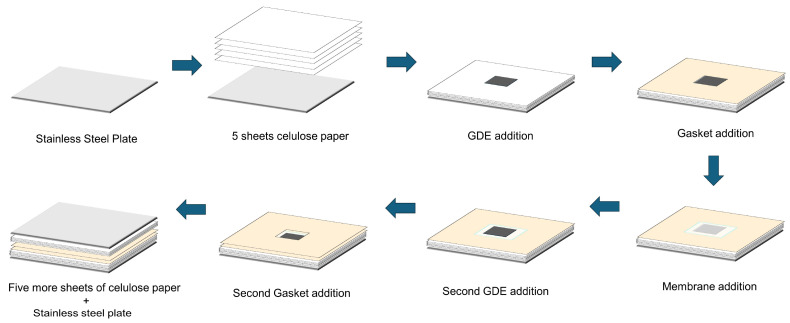
Scheme of the mounting steps employed in this work.

All the electrodes, with a geometric area of 5 cm^2^, were hot-pressed using two distinct methods. In the method referred to as ‘125C’, the electrodes were hot-pressed at 125°C for 2 min under 5 tons of pressure (normalized pressure by the electrode area was 1000 kg cm⁻^2^; normalized pressure by the stainless-steel plate area was 10 kg cm⁻^2^). Conversely, in the ‘145C’ method, the electrodes were hot-pressed at 145°C for 2 min under 0.6 tons of pressure (normalized pressure by the electrode area was 120 kg cm⁻^2^; normalized pressure by the stainless-steel plate area was 6 kg cm⁻^2^). This balance between temperature and pressure was established to prevent possible membrane damage caused by exceeding its softening temperature. During the pressing process, a 10 × 10 cm stainless steel plate was layered with five sheets of 70-micron virgin cellulose paper (grammage 20 g m^−2^). A separator made of reinforced PTFE/fiberglass (Armalon), matching the thickness of the GDE, was placed around the electrode. The membrane (area of 14 cm^2^) was positioned to fully cover the electrode surface, followed by the placement of another separator and the second electrode on the opposite side. Another five sheets of cellulose paper were then placed on top of this setup, and a second stainless steel plate of the same dimensions was added to complete the sandwich-like structure. This assembly was then transferred to a manual hot-press with electronic temperature control (Solab, SL-11) for processing the MEA. The overall area was pressed, also considering that the gasket was 100 cm^2^. The cellulose paper was introduced during the MEA to prevent the flash overheating of the membrane caused by direct contact with the metal plate and to enhance the homogeneity on the pressure distribution. The MEAs prepared in this manner were assigned acronyms as shown in Table 1, followed by ‘125C’ or ‘145C’, depending on the hot-pressing procedure used. Figure 1 shows a scheme of the step-by-step procedure. The traditional hot-pressing procedure is similar to those displayed in Figure 1, but with the absence of the cellulose paper.

### 2.4. PEMFC Test Conditions

The assembled MEA was mounted in a 5 cm^2^ commercial single cell from Fuel Cell Technologies Inc. (Albuquerque, NM, USA), consisting of two graphite plates with serpentine flow channels, secured by two metal clamping plates tightened with eight bolts. The bolts were fastened in a crosswise pattern, with the torque gradually increasing by 1 Nm at a time until the desired torque was reached. Three torque levels were tested for each cell: 9, 11, and 13 Nm. The cell was then connected to a Fuel Cell Station, the University model Fuel cell test station from Fuel Cell Technologies Inc., which provided electronic control of the cell and gas temperature, flow rate, and humidity.

The cell tightened with 9 Nm was initially preconditioned at 70 °C at 0.6 V for one hour under conditions similar to those used during measurements: O₂ and H₂ at 100% relative humidity (RH), with flow rates of 500 sccm for O₂ and 300 sccm for H₂, and the gases outlet was maintained at atmospheric pressure (ca. 0.970 atm). After preconditioning, polarization curves were recorded until consistent behavior was observed, typically requiring 5–6 measurements per cell. Before increasing the torque, the cell was cooled to room temperature to prevent interference from component dilation and as a precaution against potential membrane rupture.

Electrochemical experiments were conducted using a Potentiostat/Galvanostat Autolab PGSTAT 302N. Cyclic voltammetry (CV) measurements were performed with an Ar feed in the cathodic chamber instead of O_2_.

## 3. Results

**Figure 2:**
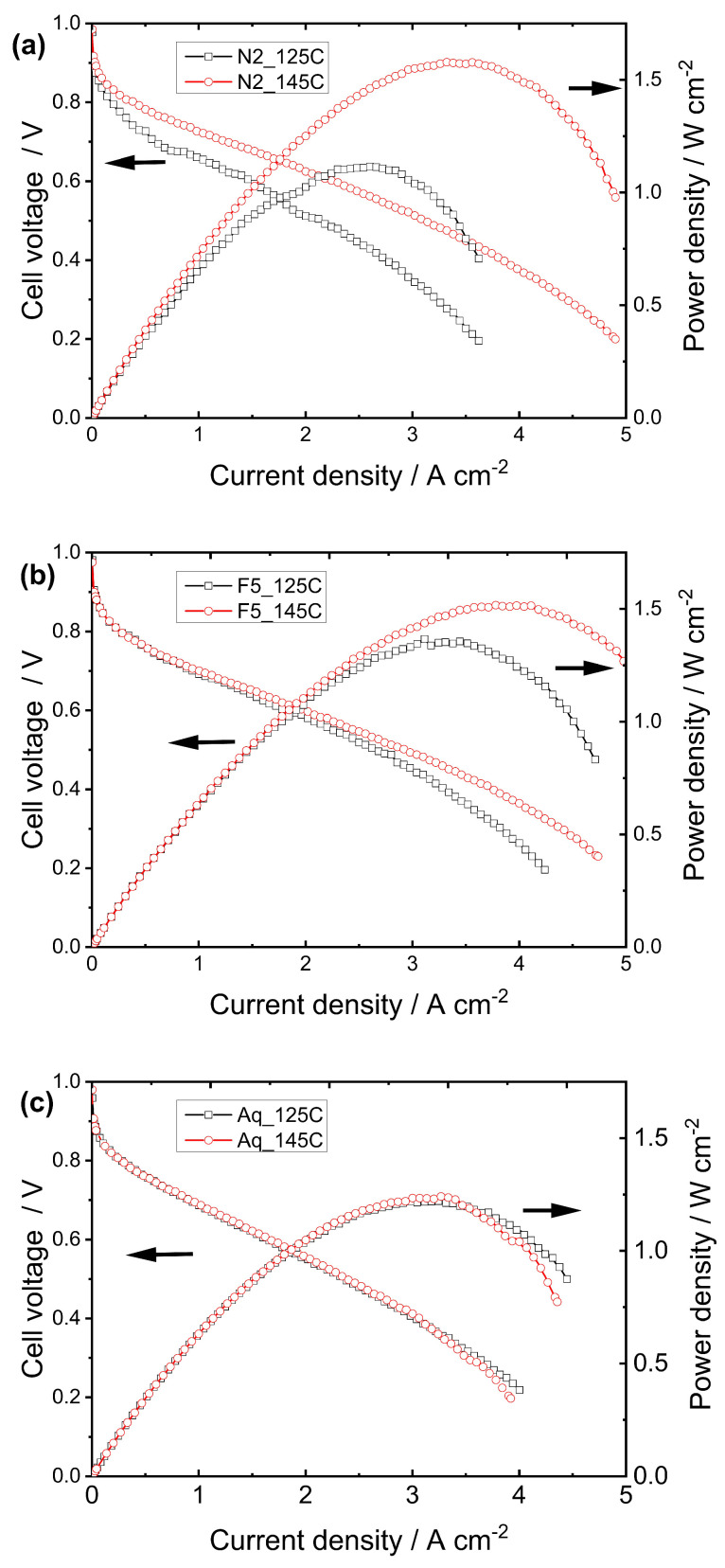
Polarization curves were acquired at 70 °C for MEAs prepared with (**a**) Nafion^®^ 212, (**b**) Fumapem F950, and (**c**) Aquivion^®^ E98-05S membranes using the 125C and 145C hot-pressing methods.

As shown in Table 1, depending on the manufacturer, each membrane has distinct characteristics that can impact fuel cell performance. To investigate factors beyond membrane thickness, an initial study was conducted to examine the effects of EW and the size of the polymer side chain. Figure 2 presents the polarization and corresponding power curves of the cells tightened at the optimal condition, all the conditions are in SI (Figures S4–S9), demonstrating that hot-pressing parameters have a significant impact on cells constructed with membranes featuring long side chains (LSC). In contrast, for membranes with short side chains (SSC), the hot-pressing method results in only a slight improvement in cell performance, with the best results obtained using the 145C method. These results can be attributed to the differences in glass transition temperatures (T_g_) between SSC and LSC membranes, with SSC membranes exhibiting higher T_g_ compared to LSC membranes [8,25,26]. Additionally, we conducted a hot-pressing experiment with the Aquivion^®^ membrane at 190 °C under 1 ton of pressure for 2 min, following the same experimental conditions applied to the other MEAs. The results are shown in Figure S1. The performance was significantly lower than that observed with the 125C and 145C methods, indicating that simply increasing the temperature is insufficient to improve catalyst utilization. Also, the decrease in the OCP and the shape of the cyclic voltammetry curve (Figure S2) indicate that hydrogen crossover dominates the process, suggesting that the membrane was damaged or that its thickness was significantly reduced during MEA preparation. Despite the difference in the membrane thickness, this finding aligns with the results of Gatto et al. [27] for Aquivion^®^ R79-02S, who found that hydrogen crossover increases as the hot-pressing temperature rises from 125 °C to 160 °C.

Another interesting feature, most evident in the Nafion and Fumapem membranes (Figure 1), is the difference in the polarization behavior of the MEAs prepared using different methods under low and high current density regimes. Both MEAs prepared with Fumapem membranes exhibit similar performance in the low to intermediate current density region. However, at currents exceeding 2.5 A cm⁻^2^, a drop in output voltage is observed. This voltage drop can be attributed to mass transfer losses caused by electrode flooding in the higher current region. On the other hand, Nafion^®^ 212 exhibits much lower cell voltages even in the low and intermediate current density regions. These findings suggest that higher pressure applied during hot-pressing reduces platinum utilization and/or the fuel availability is insufficient, likely due to the lowering of porosity caused by the ionomer content in the catalytic layer exceeding the optimal condition or insufficient Pt utilization on the cathode [24,28,29].

**Figure 3:**
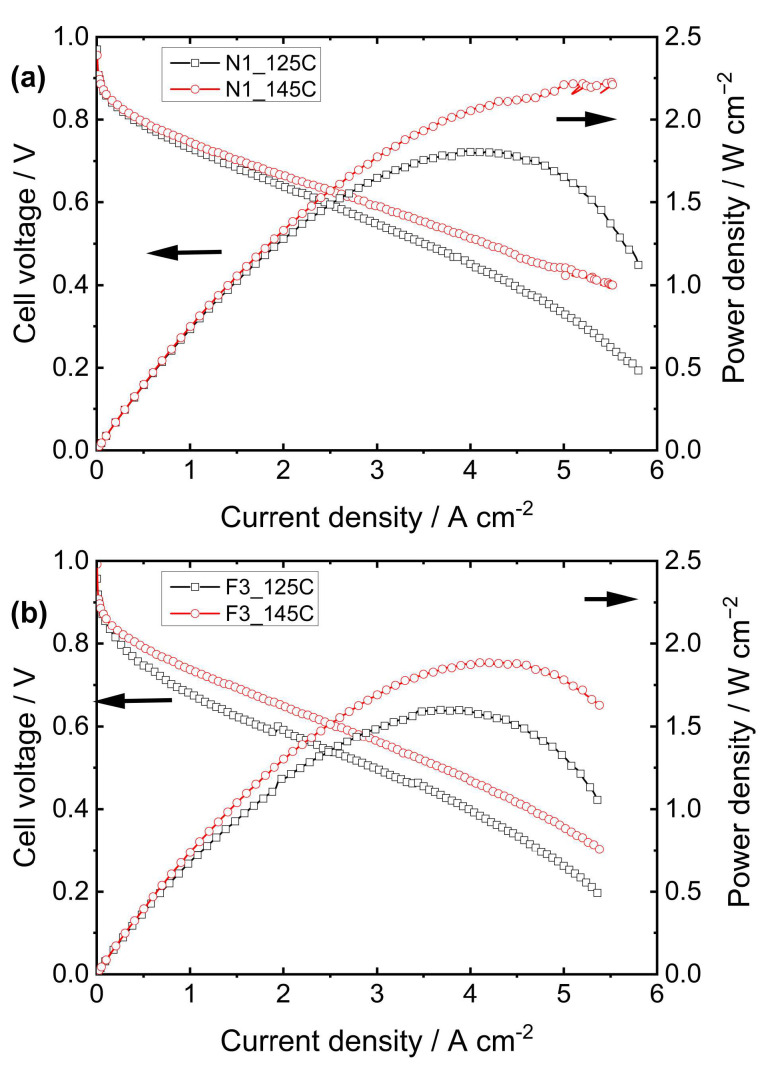
Polarization curves were acquired at 70 °C for MEAs prepared with (**a**) Nafion^®^ 211 and (**b**) Fumapem F930 membranes using the 125C and 145C hot-pressing methods.

When comparing only LSC-PFSA membranes with different EW values, it becomes evident that membranes with higher EWs (e.g., Nafion^®^ 211) are significantly more affected by hot-pressing conditions, exhibiting greater improvements in cell performance as the pressing temperature increases. These findings contrast with those reported for SSC-PFSA membranes, as LSC-PFSA membranes with lower EWs have higher T_g_ compared to those with higher EWs [30,31]. However, as pointed out by Bauer et al. [31], differences between the properties and characteristics of the ionomer in the catalytic layer and those of the membrane itself can lead to discrepancies, potentially influencing overall performance. The commercial GDE used in our studies contains approximately 0.4 mg cm⁻^2^ of Pt, but no specific details regarding the type of PFSA ionomer employed are provided. For thinner membranes, as shown in Figure 3, the polarization curves (Nafion^®^ 211 and Fumapem F930) reveal behavior that is similar to that observed in membranes with a thickness of around 50 microns, where membranes with higher EWs exhibit greater performance improvements as the pressing temperature increases. It is important to note that Nafion^®^ 211 is somewhat thinner than Fumapem F930, contributing to its improved cell performance since thinner membranes result in a lower internal resistance.

Similarly to the behavior observed in Figure 2a,b, the polarization curves of thinner membranes pressed in the 125C (pressed at 5 ton) and 145C (pressed at 0.6 ton) methods also displayed a strong dependence on current density, with the 145C method yielding significantly higher performance compared to the 125C method. However, only a slight difference was observed for the Aquivion^®^ membrane (Figure 2c). This suggests that LSC membranes are more sensitive to temperature than SSC membranes, which aligns with their lower Tg and greater susceptibility to thermal effects. In the study by Okur et al. [17], which evaluated twenty hot-pressed MEAs prepared by 15 different methods using Nafion^®^ 212, it was found that at low temperatures (80 °C) and higher applied pressure (100 kg cm⁻^2^), the fuel cell performance was slightly lower than when the pressure was reduced to 55 kg cm⁻^2^. However, when 100 kg cm⁻^2^ and 130 °C were applied, the maximum power density decreased by approximately 130 mW cm⁻^2^, suggesting that membrane softening and high pressure likely hindered platinum active sites, diminishing overall MEA performance. Conversely, by employing the same temperature but much lower pressure (10 kg cm⁻^2^), the MEA performance was far from optimal, displaying a significant decrease of 320 mW cm⁻^2^.

Lin and collaborators [15] further investigated the effect of MEA preparation temperature (using Nafion^®^ 112) under a fixed pressure of 50 kg cm⁻^2^. They demonstrated that at 150 °C, both platinum utilization and hydrogen crossover were strongly increased, whereas optimal conditions were achieved at 125 °C. Similarly, Martemianov et al. [18] investigated the impact of hot-pressing on Nafion^®^ 112, finding the optimal MEA performance when employing a temperature of 100 °C and a pressure of 56 kg cm⁻^2^ for pressing electrodes with a 5 cm^2^ active area.

Regarding SSC-PFSA membranes, Gatto et al. [27] investigated the effects of hot-pressing at temperatures of 125 °C and 160 °C at a pressure of 25 kg cm⁻^2^ on Aquivion^®^ R79-02S membranes. Despite their results being obtained at 50% RH, the highest fuel cell performance was achieved for MEAs prepared at 125 °C. However, their study focused solely on temperature as a variable and did not explore the effect of pressure on MEA performance, leaving this aspect unaddressed.

**Figure 4:**
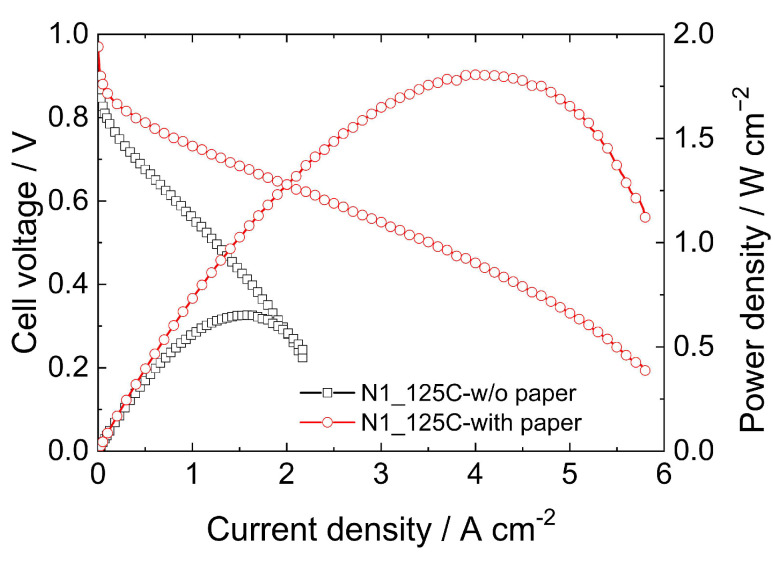
Polarization curves acquired at 70 °C for MEAs prepared with Nafion^®^ 211 using the 125C method with (red curve) and without (black curve) employing cellulose paper in the pressing set.

Most of the previously reported studies demonstrate that hot-pressing at temperatures around 125 °C is the best option for preparing MEAs, independent of the side chain length or equivalent weight. However, detailed descriptions of the assembly process and the area over which the applied pressure is distributed are often lacking when examining the specific procedures related to the pressing step. Considering the area of the entire setup positioned in the pressing machine (100 cm^2^), as noted in previously reported works from our group [24,32], the applied pressure drops to only a few kilograms per centimeter squared, as outlined in the Materials and Methods Section. The pressure values employed in our work fall below the pressures used in the cited studies, but the most notable difference is the use of a pressure distributor. Figure 4 displays the polarization curve and corresponding power density of an MEA prepared with Nafion^®^ 211 without the cellulose papers used to improve pressure distribution, compared to the MEA prepared with the cellulose paper. To the best of our knowledge, this is the first time such parameters in hot-pressing methods have been reported and discussed, opening new opportunities for enhancing the performance of PEMFCs. Additionally, to improve the repeatability and reproducibility of reported data, it is highly recommended to include detailed information about the hot-pressing method, such as assembly specificity and pressure distribution. The results indicate that significantly lower performance is obtained when cellulose paper is not included in the pressing setup, leading to lower voltages at a given current density and a decrease in the OCP. Comparing the maximum power densities obtained by both methods, the MEA prepared with cellulose paper achieved 1.82 W cm⁻^2^, whereas the MEA prepared without cellulose paper achieved only 0.66 W cm⁻^2^. Notably, our results for the MEA prepared without cellulose paper are in the range of those reported in the literature [33,34,35,36]. However, to the best of our knowledge, when cellulose paper is included, no prior studies on hot-pressing catalyst-coated GDEs have surpassed the performance reported here. Moreover, the 145C method demonstrated a maximum power density of approximately 2.2 W cm⁻^2^, further highlighting the effectiveness of the optimized process.

The effect of the hot-pressing method on platinum utilization was investigated by cyclic voltammetry with Ar-feed in the cathodic chamber and was performed for the 145C MEAs prepared with Nafion^®^, Fumapem, and Aquivion^®^ membranes. Figure S3 shows typical cyclic voltammogram shapes expected for a polycrystalline platinum electrode for all prepared MEAs. The current densities observed in the hydrogen adsorption/desorption region on the CV are similar across all prepared MEAs, indicating that the catalytic layers in all MEAs prepared using the 145C method contain comparable platinum surface areas. These results may appear to conflict with the performance differences observed in the polarization curves shown in Figure 2, where the MEAs displayed varying performances depending on the membrane type. However, it is important to note that under Ar-feed conditions, a two-phase interaction between the Pt particles and the electrolyte (ionomer) is sufficient to produce the electrochemical response. In contrast, when the cell is fed with oxygen, a functional triple-phase boundary is required. In this sense, modifications to the Pt surface area would be expected only if there were changes in particle size [37] or the leaching of the metal catalyst from the carbon support [11]. The insert in Figure S3 presents the hydrogen desorption charge values obtained under the same conditions as the curves in Figure 2. These data reveal a general trend of higher hydrogen desorption charges for MEAs prepared using the 125C method, indicating that Pt coalescence is more prone to occur at higher hot-pressing temperatures, in agreement with the results reported by Liang et al. [37]. Despite this, the superior fuel cell performances obtained by employing the 145C method for MEA preparation demonstrate that factors other than platinum surface area play a more critical role in the performance improvement. Based on the presented results, it is clear that the 125C method exhibits worse performance compared to the 145C method. This is expected due to the higher pressure applied during the hot-pressing step in the 125C method, as the CV results indicate a higher platinum surface area for MEAs prepared using this method. Regarding the application of higher pressures in hot-pressing methods, several effects may contribute to the observed performance degradation: (1) higher compression can deform the carbon paper, reducing its gas permeability and hindering effective reactant transport; (2) excessive pressure can lead to the deformation of the microporous layer, resulting in a non-homogeneous distribution of fuel to the catalytic layer; (3) the PFSA from the membrane may penetrate into the catalytic layer, covering platinum active sites and hindering their accessibility; (4) high pressure can alter the nano-domain structure of PFSA, potentially affecting ion transport and overall MEA performance. Despite these possible issues, further investigations using cutting-edge techniques, such as X-ray computed tomography, will be conducted to reconstruct and analyze the interface created by attaching a GDE to the membrane.

The effect of the torque applied to the cell assembly was also evaluated, and all polarization curves obtained are shown in Figures S4–S13. Table 2 summarizes the OCP and current density at 0.75 V (i@0.75V) for the cell. This voltage was selected as it approximates 50% energy efficiency based on the thermoneutral potential (1.482 V) [38], (DH/nF, where DH is the enthalpy of water formation) which accounts for the combined the electrical work and heat produced by the reactions occurring in the PEMFC. Only the MEA Aq_125C displayed no significant impact from cell tightening for all experiments, showing consistent i@0.75V and OCP values across all torque conditions. In contrast, all other MEAs demonstrated variability in performance, with no clear trend between increasing bolt torque and i@0.75V. However, the OCP values for MEAs prepared with the 125C method were often lower than those prepared with the 145 °C method. A particularly notable difference was observed for the N1_145C MEA, which exhibited the lowest OCP values and a significant drop when the torque was increased to 13 Nm, indicating increased hydrogen crossover. Examining the polarization curves from intermediate to higher current densities, it is evident that cell tightening has varying effects depending on the membrane and MEA preparation process. For instance, Aq_125C (Figure S4), which showed consistent figures of merit in Table 2, revealed a slight potential decrease at higher current densities as the bolt torque increased, indicative of mass transport losses. On the other hand, Aq_145C exhibited diminished current densities as the cell tightness increased. Other performance-limiting factors (possibly a smaller Pt active surface area) are suggested even at low current densities. This behavior resembles findings by Passalacqua et al. [22] in a fuel cell constructed with reinforced nitrile rubber (NBR) as a gasket. Interestingly, when Passalacqua et al. employed a PTFE gasket—a material with a high Young’s modulus and low deformability—the polarization curves remained almost unchanged as bolt torque increased. Using a pressure sensor, they observed that as bolt torque increased, pressure distribution near the center of the electrode tended to create a low-compression zone. This phenomenon was attributed to the deformation of the GDE caused by endplate distortion during tightening, highlighting the critical role of uniform pressure distribution in MEA performance. For the other membranes, Fumapem and Nafion^®^, bolt torque exhibited no clear correlation with cell performance. In the case of Fumapem membranes with greater thickness, higher bolt torques improved performance for MEAs prepared using the 125C method. Conversely, for F5_145C MEA, lower bolt torques yielded better performance. When the thickness of the Fumapem membranes was reduced, cell tightening strongly influenced performance for MEAs prepared with the 125C method. However, for the 145C method, performance remained nearly constant across all tightening conditions. Similarly, regardless of the preparation method, Nafion^®^-based MEAs showed no discernible trend linking bolt torque to cell performance. These findings suggest that the interplay between cell performance and MEA methods is highly complex, depending on several factors such as the type of gasket employed, cell tightness, GDE deformability, and others.

Although the prepared MEAs were not subjected to durability studies, the OCP serves as a key figure of merit for assessing potential degradation. For instance, the N1_145C membrane exhibits the lowest OCP values, a trend that becomes more pronounced at higher tightening torques. This suggests that hydrogen crossover is more likely to occur when the MEA is prepared using this method. It is well known that hydrogen crossover can significantly impact cell durability, not only by increasing fuel loss but also by accelerating catalyst degradation. Specifically, during start/stop conditions, the dissolution of Pt ions is enhanced, and these dissolved species can migrate from the anode to the cathode [39]. The presence of hydrogen facilitates their reduction, leading to Pt nanoparticle growth or agglomeration [40]. Additionally, the incorporation of solid particles into the membrane can reduce its ionic conductivity, further compromising overall fuel cell performance and longevity [41].

It is also worth noting that under the operating conditions tested at high current densities, no deficiencies related to water management were observed. However, while this is not the focus of the present study, effective water management becomes increasingly crucial for optimizing the long-term performance and durability of PEMFCs. Although the membrane serves as the primary water transporter and influences water management, the properties of the GDE, such as hydrophobicity, pore volume distribution, MPL characteristics, and catalytic layer distribution, also play a crucial role in water drainage. However, a commercial GDE was chosen in this study to minimize the number of variables and ensure a more controlled evaluation of the investigated parameters. The large-scale commercialization of PEMFCs requires higher power and current densities, but excessive liquid water accumulation in the gas diffusion layer (GDL) can lead to flooding, obstruct gas transport, and significantly degrade cell performance [42,43].

It is important to note that our results do not aim to determine which membrane is superior, as the same MEA study protocol was applied consistently to all membranes. While SSC membranes are often recommended for operation at higher temperatures (>90 °C), LSC membranes remain the most extensively studied in the literature [44]. However, their lower T_g_ limits their applicability at elevated temperatures.

Therefore, this work demonstrates that hot-pressing methods for producing MEAs play a critical role in fuel cell performance. More importantly, it highlights the necessity of reporting detailed experimental procedures to ensure the reproducibility and repeatability of fuel cell performance. As noted, many previously published studies lack comprehensive descriptions of MEA methods, which appears to be a common oversight among researchers in this field. To address this, it is recommended that future studies provide detailed reports of all steps involved in MEA preparation, including the thickness, characteristics, and size of the separators, their positioning, and assembly, as well as the membrane and electrode dimensions and other factors that may influence electrode assembly.

A particularly critical parameter identified in this study is the pressure homogeneity during hot-pressing. Commonly used gaskets often exhibit non-uniform thicknesses, and membranes may develop wrinkled surfaces after activation. These defects can create regions of overpressure during the pressing process, leading to poor electrode attachment and ultimately hindering the full performance potential of the fuel cell. It is also worth noting that further studies are necessary to conduct an in-depth investigation of the membrane|GDE interface.

## 4. Conclusions

In this study, MEAs were prepared using commercial GDEs, membranes with different characteristics (side chain size and equivalent weight), and two hot-pressing methods, and their performance in PEMFCs was investigated under varying tightening conditions. The hot-pressing methods played a critical role in MEA performance, with the method applying a higher temperature but lower pressure (145C method) yielding superior current and power densities. PFSA membranes with long side chains (LSCs) exhibited better fuel cell performance, which was attributed to their lower glass transition temperature compared to short side chain (SSC) membranes. Additionally, the use of cellulose papers as a pressure homogenizer during the hot-pressing process significantly enhanced MEA performance, improving it by approximately 2.5 times compared to the traditional method that does not employ this feature. The active surface area of platinum was nearly identical for MEAs prepared with different membranes but using the same hot-pressing method, indicating that fuel cell performance is more sensitive to pressure than temperature. The cell tightening, controlled by bolt torque, exhibited almost no consistent trend in cell performance across MEAs constructed with different methods.

Overall, MEAs constructed using the 145C method performed better, with those assembled using thinner membranes achieving higher current densities across both low and high current density regions compared to thicker membranes. Conversely, thicker membranes displayed higher OCP values, mainly when the 145C method was employed for MEA preparation. Finally, this study demonstrates the significant influence of membrane characteristics and hot-pressing conditions on fuel cell performance, providing valuable insights for optimizing MEA preparation in PEMFCs.

## Figures and Tables

**Table 1:** Summary of the membrane characteristics according to each manufacturer.

Membrane	Thickness in Dry Form/µm	EW/g eq^−1^	Side Chain Length	Sample Acronym
Nafion 211	25.4	1100	Long	N1
Nafion 212	50.8	1100	Long	N2
Fumapem F-930	30	900	Long	F3
Fumapem F-950	50	900	Long	F5
Aquivion E98-05S	50	980	Short	Aq

**Table 2:** Current density (A cm^−2^) at cell potential of 0.75 V and open circuit potential/V for different membrane electrode assembly studies.

Membrane	Current Density (A cm^−2^) at Cell Potential of 0.75 V			Open Circuit Potential/V		
	Cell Tightening/Nm			Cell Tightening/Nm		
	9	11	13	9	11	13
Aq_125C	0.567	0.567	0.567	0.97	0.97	0.97
Aq_145C	0.550	0.565	0.347	0.98	1.00	0.98
F5_125C	0.273	0.311	0.541	0.96	0.97	0.99
F5_145C	0.568	0.593	0.367	0.99	1.00	0.99
N2_125C	0.272	0.351	0.351	0.99	0.99	1.00
N2_145C	0.509	0.534	0.771	0.99	1.00	1.00
F3_125C	0.453	0.489	0.329	0.98	0.98	1.00
F3_145C	0.834	0.802	0.865	0.98	0.98	1.00
N1_125C	0.740	0.825	0.838	0.97	0.98	1.00
N1_145C	0.842	0.940	0.699	0.97	0.97	0.92

## Data Availability

Data are available from the corresponding author upon reasonable request.

## Supplementary Materials

The following supporting information can be downloaded at: https://www.mdpi.com/article/10.3390/membranes15030065/s1, Figure S1: Polarization curves acquired at 70 °C for MEAs prepared with Aquivion^®^ E98-05S membranes, hot-pressed at 190 °C under 1 ton of pressure for 2 min; Figure S2: Cyclic voltammogram acquired at 20 mV s⁻^1^ at room temperature for MEAs prepared with Aquivion^®^ E98-05S membranes, hot-pressed at 190 °C under 1 ton of pressure for 2 min; Figure S3: Cyclic voltammogram acquired at 50 mV s⁻^1^ at room temperature for MEAs prepared with Aquivion^®^ E98-05S, Fumapem F950, and Nafion^®^ 212 membranes, hot-pressed at 145 °C under 0.6 ton of pressure for 2 min; Figure S4: Polarization curves acquired at 70 °C for MEAs prepared with Aquivion^®^ E98-05S membranes, hot-pressed at 125 °C under 5 tons of pressure for 2 min; Figure S5: Polarization curves acquired at 70 °C for MEAs prepared with Aquivion^®^ E98-05S membranes, hot-pressed at 145 °C under 0.6 ton of pressure for 2 min; Figure S6: Polarization curves acquired at 70 °C for MEAs prepared with Fumapem F950 membranes, hot-pressed at 125 °C under 6 tons of pressure for 2 min; Figure S7: Polarization curves acquired at 70 °C for MEAs prepared with Fumapem F950 membranes, hot-pressed at 145 °C under 0.6 ton of pressure for 2 min; Figure S8: Polarization curves acquired at 70 °C for MEAs prepared with Nafion^®^ 212 membranes, hot-pressed at 125 °C under 5 tons of pressure for 2 min; Figure S9: Polarization curves acquired at 70 °C for MEAs prepared with Nafion^®^ 212 membranes, hot-pressed at 145 °C under 0.6 ton of pressure for 2 min; Figure S10: Polarization curves acquired at 70 °C for MEAs prepared with Fumapem F930 membranes, hot-pressed at 125 °C under 5 tons of pressure for 2 min; Figure S11: Polarization curves acquired at 70 °C for MEAs prepared with Fumapem F930 membranes, hot-pressed at 145 °C under 0.6 ton of pressure for 2 min; Figure S12: Polarization curves acquired at 70 °C for MEAs prepared with Nafion^®^ 211 membranes, hot-pressed at 125 °C under 5 tons of pressure for 2 min; Figure S13: Polarization curves acquired at 70 °C for MEAs prepared with Nafion^®^ 211 membranes, hot-pressed at 145 °C under 0.6 ton of pressure for 2 min.
