# Supplementary material for: Uncovering Key Parameters in Perfluorosulfonic Acid (PFSA) Membrane Fuel Cells to Enhance Performance
Source: Membranes (Basel). 2025 Feb 20;15(3):65. doi: 10.3390/membranes15030065 (PMC11944044; doi:10.3390/membranes15030065)
Supplement: Supplementary file 1 [file membranes-15-00065-s001.zip › membranes-3445475-supplementary.pdf]

Uncovering key parameters in Perfluorosulfonic Acid (PFSA) Membrane Fuel Cells to enhance performance

Valdecir Antonio Paganin[1], Alan M. P. Sakita[1], Thiago Lopes[2] Edson Antonio Ticianelli[1], Joelma Perez[1,*]

[1] University of São Paulo, São Carlos Institute of Chemistry. 13560-970, São Paulo, Brazil;vpaganin@iqsc.usp.br, ampsakita@gmail.com, thiago_lopes@usp.br, edsont@iqsc.usp.br

[2] University of São Paulo, Department of Mechatronics and Mechanical Systems Engineering of Escola Politecnica, 05508-030, São Paulo, Brazil; thiago_lopes@usp.br

* Correspondence: jperez@iqsc.usp.br

Table of Contents

Figure S1. Polarization curves acquired at 70°C for MEAs prepared with Aquivion® E98-05S membranes, hot-pressed at 190°C under 1 ton of pressure for 2 minutes. 2

Figure S2. Cyclic voltammogram acquired at 20 mV s^{-1} at room temperature for MEAs prepared with Aquivion® E98-05S membranes, hot-pressed at 190°C under 1 ton of pressure for 2 minutes...2

Figure S3. Cyclic voltammogram acquired at 50 mV s^{-1} at room temperature for MEAs prepared with Aquivion® E98-05S, Fumapem F950, and Nafion® 212 membranes, hot-pressed at 145°C under 0.6 ton of pressure for 2 minutes. 3

Figure S4. Polarization curves acquired at 70°C for MEAs prepared with Aquivion® E98-05S membranes, hot-pressed at 125°C under 5 ton of pressure for 2 minutes. 3

Figure S5. Polarization curves acquired at 70°C for MEAs prepared with Aquivion® E98-05S membranes, hot-pressed at 145°C under 0.6 ton of pressure for 2 minutes. 4

Figure S6. Polarization curves acquired at 70°C for MEAs prepared with Fumapem F950 membranes, hot-pressed at 125°C under 6 ton of pressure for 2 minutes. 4

Figure S7. Polarization curves acquired at 70°C for MEAs prepared with Fumapem F950 membranes, hot-pressed at 145°C under 0.6 ton of pressure for 2 minutes. 5

Figure S8. Polarization curves acquired at 70°C for MEAs prepared with Nafion® 212 membranes, hot-pressed at 125°C under 5 ton of pressure for 2 minutes. 5

Figure S9. Polarization curves acquired at 70°C for MEAs prepared with Nafion® 212 membranes, hot-pressed at 145°C under 0.6 ton of pressure for 2 minutes. 6

Figure S10. Polarization curves acquired at 70°C for MEAs prepared with Fumapem F930 membranes, hot-pressed at 125°C under 5 ton of pressure for 2 minutes. 6

Figure S11. Polarization curves acquired at 70°C for MEAs prepared with Fumapem F930 membranes, hot-pressed at 145°C under 0.6 ton of pressure for 2 minutes. 7

Figure S12. Polarization curves acquired at 70°C for MEAs prepared with Nafion® 211 membranes, hot-pressed at 125°C under 5 ton of pressure for 2 minutes. 7

Figure S13. Polarization curves acquired at 70°C for MEAs prepared with Nafion® 211 membranes, hot-pressed at 145°C under 0.6 ton of pressure for 2 minutes. 8

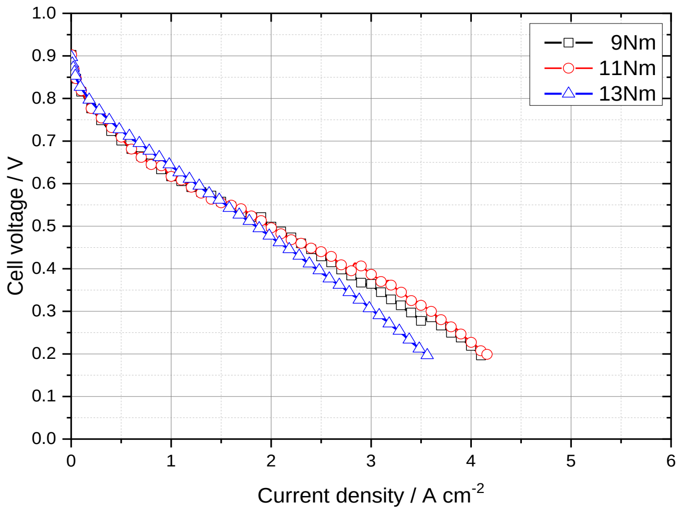

Figure S1. Polarization curves acquired at 70°C for MEAs prepared with Aquivion® E98-05S membranes, hot-pressed at 190°C under 1 ton of pressure for 2 minutes.

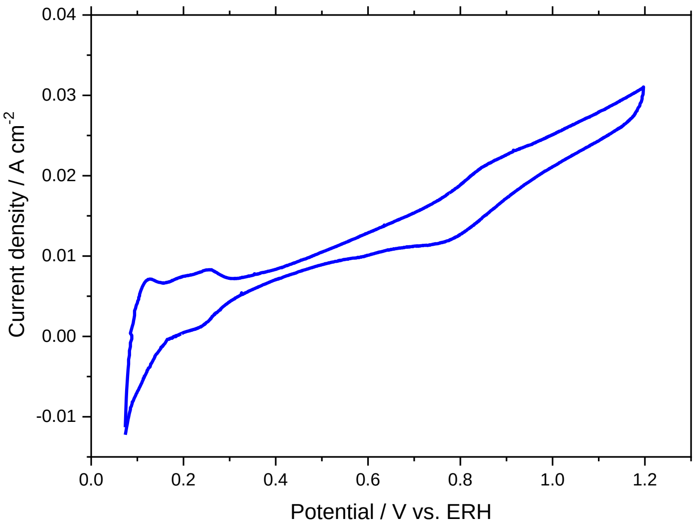

Figure S2. Cyclic voltammogram acquired at 20 mV s^{-1} at room temperature for MEAs prepared with Aquivion® E98-05S membranes, hot-pressed at 190°C under 1 ton of pressure for 2 minutes.

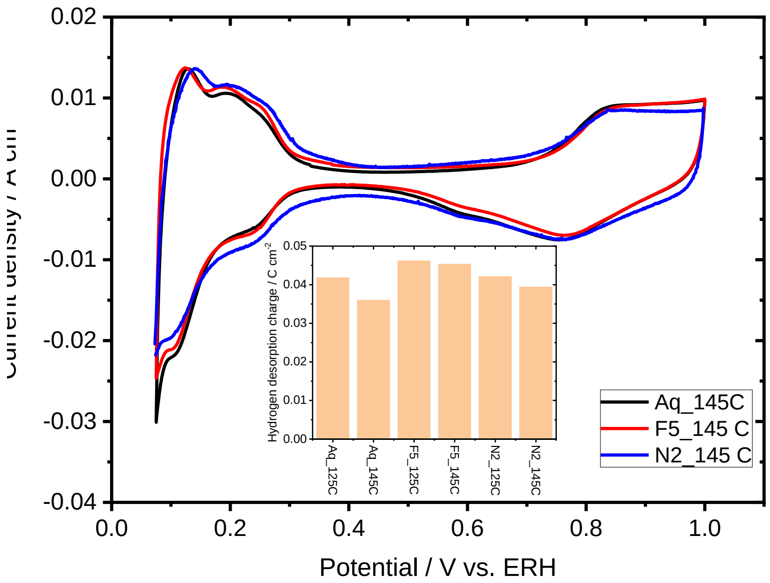

Figure S3. Cyclic voltammogram acquired at 50 mV s^{-1} at room temperature for MEAs prepared with Aquivion® E98-05S, Fumapem F950, and Nafion® 212 membranes, hot-pressed at 145°C under 0.6 ton of pressure for 2 minutes. Insert: Hydrogen adsorption charge.

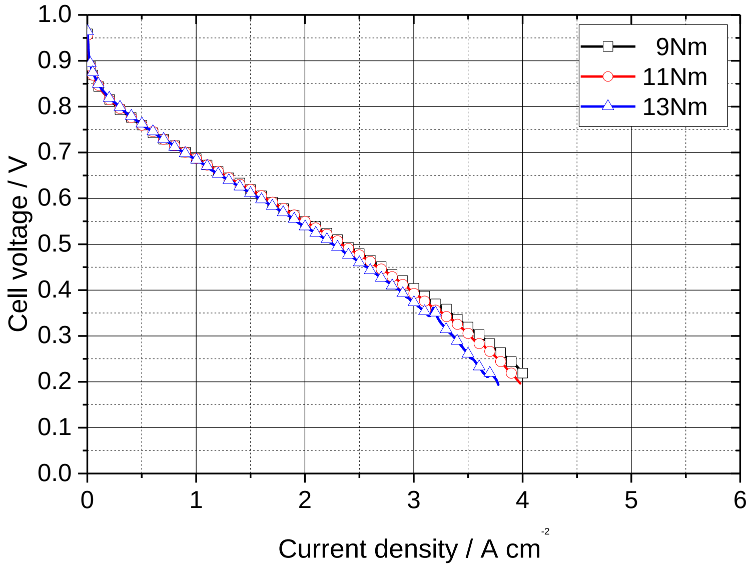

Figure S4. Polarization curves acquired at 70°C for MEAs prepared with Aquivion® E98-05S membranes, hot-pressed at 125°C under 5 ton of pressure for 2 minutes.

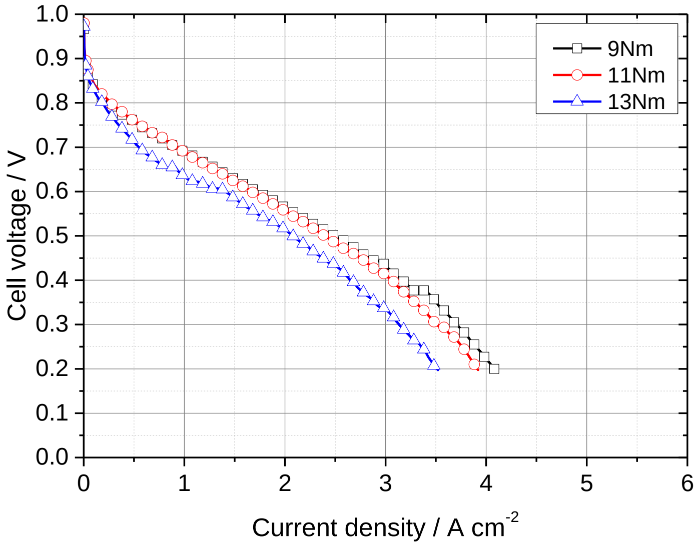

Figure S5. Polarization curves acquired at 70°C for MEAs prepared with Aquivion® E98-05S membranes, hot-pressed at 145°C under 0.6 ton of pressure for 2 minutes.

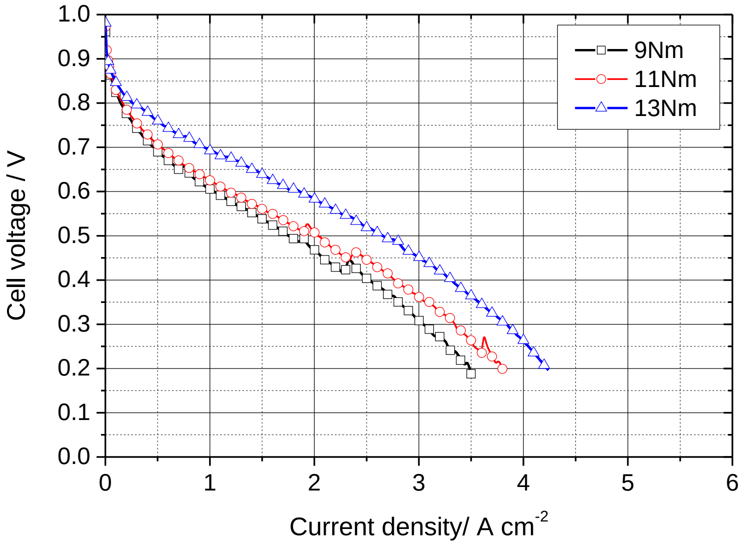

Figure S6. Polarization curves acquired at 70°C for MEAs prepared with Fumapem F950 membranes, hot-pressed at 125°C under 6 ton of pressure for 2 minutes.

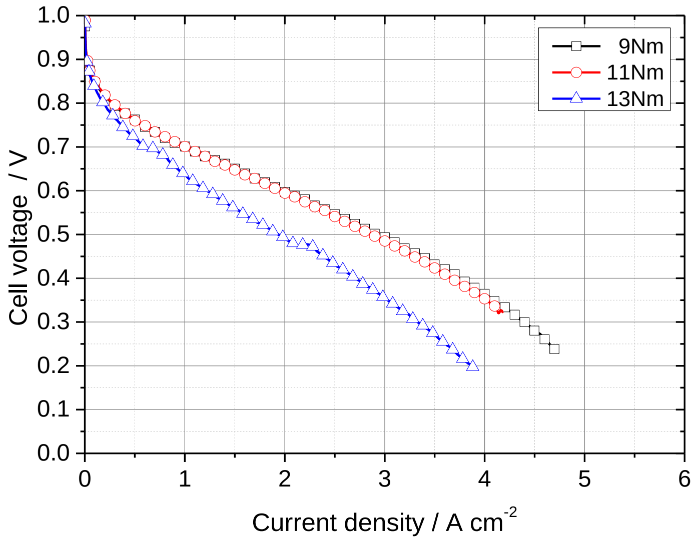

Figure S7. Polarization curves acquired at 70°C for MEAs prepared with Fumapem F950 membranes, hot-pressed at 145°C under 0.6 ton of pressure for 2 minutes.

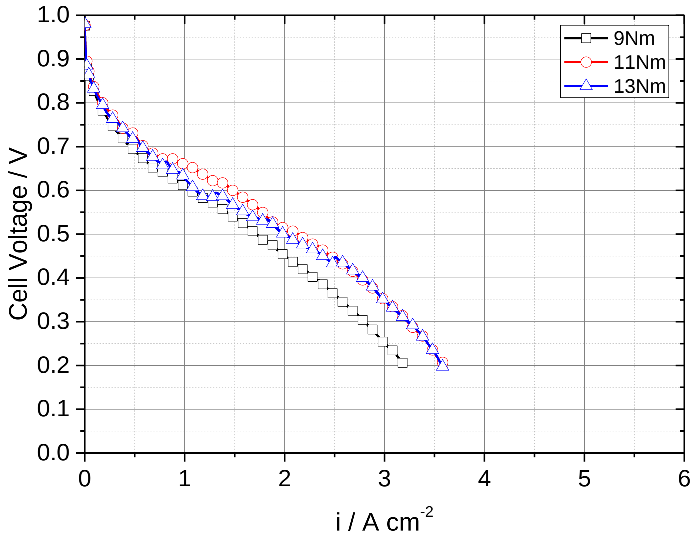

Figure S8. Polarization curves acquired at 70°C for MEAs prepared with Nafion® 212 membranes, hot-pressed at 125°C under 5 ton of pressure for 2 minutes.

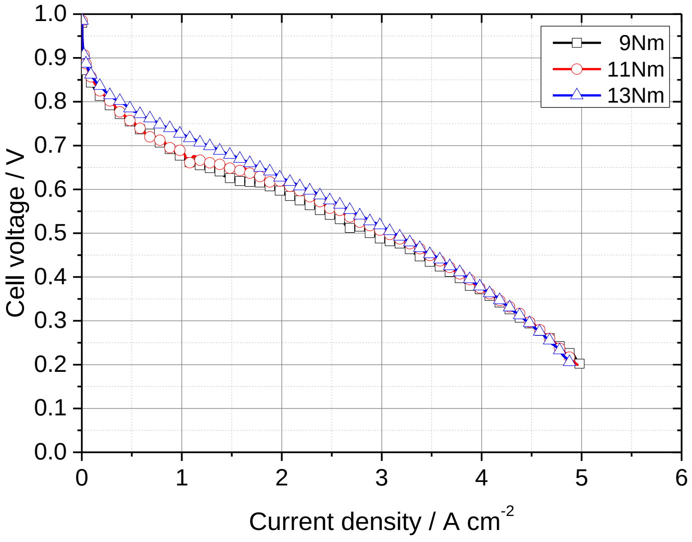

Figure S9. Polarization curves acquired at 70°C for MEAs prepared with Nafion® 212 membranes, hot-pressed at 145°C under 0.6 ton of pressure for 2 minutes.

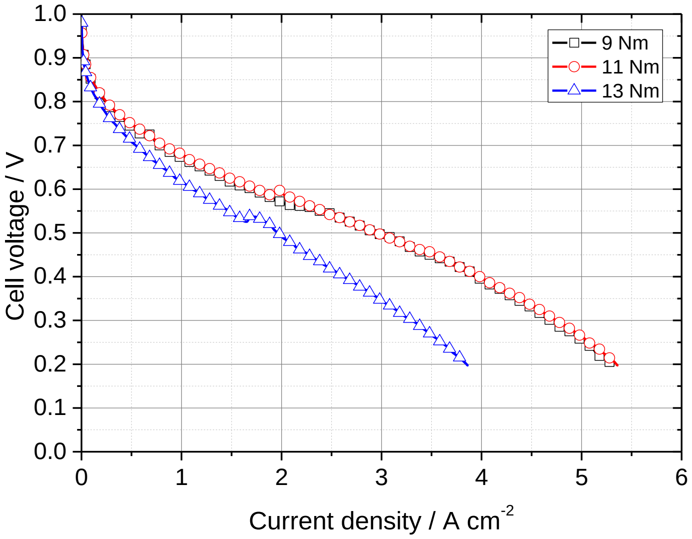

Figure S10. Polarization curves acquired at 70°C for MEAs prepared with Fumapem F930 membranes, hot-pressed at 125°C under 5 ton of pressure for 2 minutes.

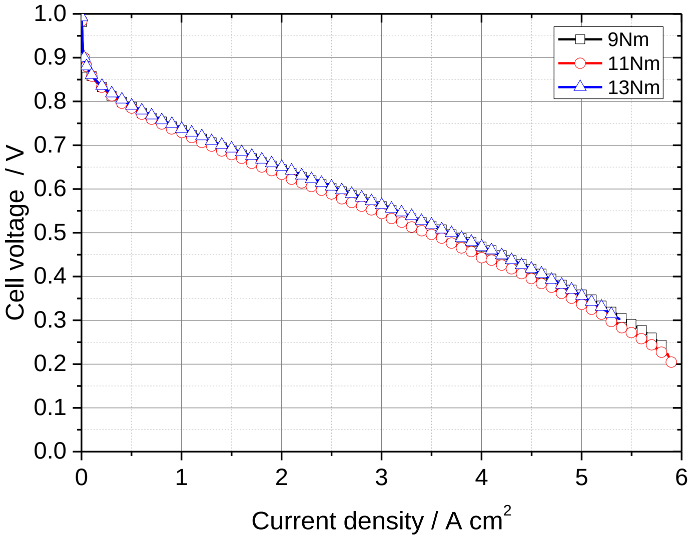

Figure S11. Polarization curves acquired at 70°C for MEAs prepared with Fumapem F930 membranes, hot-pressed at 145°C under 0.6 ton of pressure for 2 minutes.

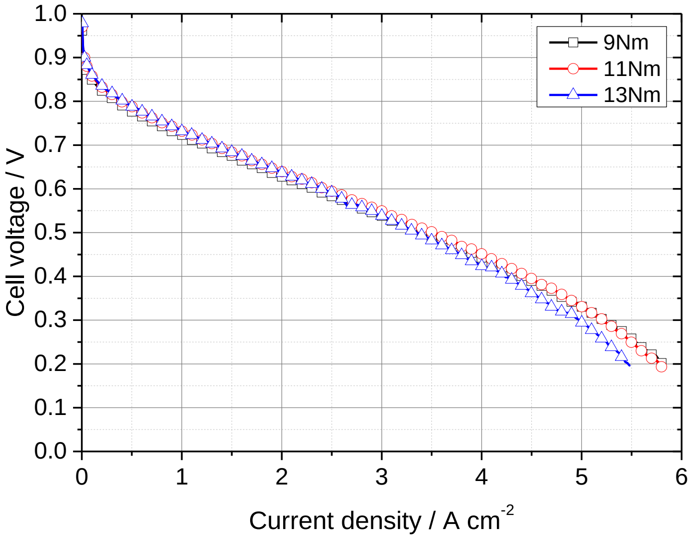

Figure S12. Polarization curves acquired at 70°C for MEAs prepared with Nafion® 211 membranes, hot-pressed at 125°C under 5 ton of pressure for 2 minutes.

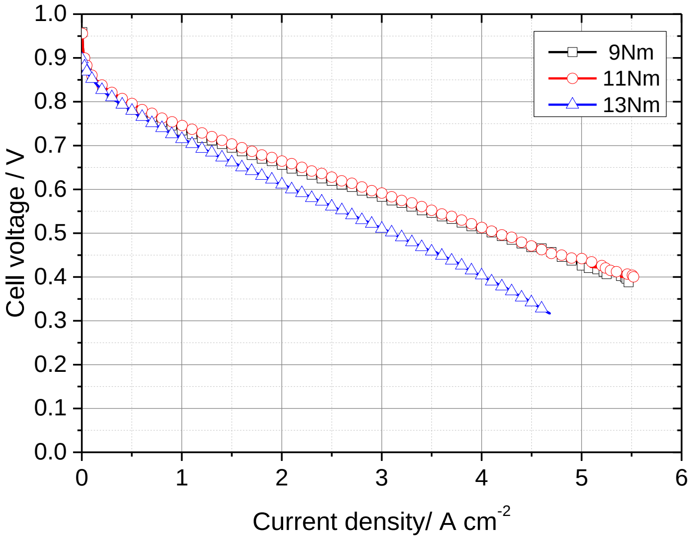

Figure S13. Polarization curves acquired at 70°C for MEAs prepared with Nafion® 211 membranes, hot-pressed at 145°C under 0.6 ton of pressure for 2 minutes.
